# Examining the Influence of Demographic and Socioeconomic Factors on Disparities in Health Care App Usage: Protocol for a Systematic Scoping Review

**DOI:** 10.2196/63596

**Published:** 2025-07-15

**Authors:** Fahad Aljuaid, Emily Reed, Sara Imanpour, Daniel J Mallinson

**Affiliations:** 1 Penn State Harrisburg Middletown, PA United States

**Keywords:** access to primary care, digital health, eHealth, health service accessibility, mHealth, mobile health applications, technology acceptance, telemedicine, demographic, socioeconomic factor, disparities, health care app, app usage, protocol, systematic review, medical information, health monitoring, health outcomes, effectiveness

## Abstract

**Background:**

The rapid proliferation of health care apps has transformed health care delivery, providing patients with unprecedented access to medical information and services. These apps facilitate remote consultations, appointment scheduling, medication reminders, and health monitoring, thereby enhancing patient engagement and improving health outcomes. Despite the widespread benefits, disparities in the adoption and usage of health care apps persist, influenced by demographic and socioeconomic factors. Understanding these disparities is crucial for designing interventions that promote equitable access to digital health tools.

**Objective:**

This systematic review aims to identify and synthesize empirical studies on health care app usage disparities, focusing on demographic and socioeconomic factors. This review seeks to inform stakeholders about the key factors influencing app usage and provide insights to improve accessibility and effectiveness. Specifically, this review addresses the following research questions: (1) what are the key demographic and socioeconomic factors associated with health care app usage disparities? (2) how do these factors influence the adoption and utilization of health care apps? and (3) what are the barriers to and facilitators of effective use of health care apps?

**Methods:**

This review will adhere to PRISMA-P (Preferred Reporting Items for Systematic Review and Meta-Analysis Protocols) guidelines. Eight databases—ACM Digital Library, CINAHL, IEEE Xplore, ProQuest Nursing and Allied Health Journals, PubMed/MEDLINE, ScienceDirect, Scopus, and Web of Science—were searched for studies published in English between January 1, 2014, and June 17, 2024. Eligibility criteria include journal papers focusing on health care app usage across different demographic and socioeconomic groups. Data management will involve using Zotero for reference management and Excel for screening and eligibility assessment. Two reviewers will independently extract the data and assess the study quality and risk of bias. Descriptive statistics will be used to summarize the study characteristics.

**Results:**

As of June 2025, the review is in the screening stage. The completion of data collection is anticipated by November 2025. The final results are expected to be published by late 2025. This review aims to provide comprehensive insights into the disparities in health care app usage.

**Conclusions:**

The findings of this systematic review will offer valuable insights into demographic and socioeconomic disparities in health care app usage, informing stakeholders on how to address these disparities. By identifying the factors influencing app adoption and usage, this review will contribute to the development of targeted interventions and policies to enhance digital health equity.

**International Registered Report Identifier (IRRID):**

DERR1-10.2196/63596

## Introduction

### Rationale

The rapid proliferation of health care apps has significantly transformed health care delivery, providing patients with unprecedented access to medical information and services [1,2]. These apps facilitate remote consultations, appointment scheduling, medication reminders, and health monitoring, thereby enhancing patient engagement and improving health outcomes. The convenience and accessibility offered by health care apps are especially crucial in managing chronic diseases and ensuring continuity of care, particularly in remote and underserved areas [3].

Despite the widespread benefits, the adoption and usage of health care apps are not uniform across different population groups. Previous studies have identified significant disparities in pursuing health-related information online based on demographic and socioeconomic factors [4]. These disparities can exacerbate health inequities, as certain groups may lack the necessary resources or digital literacy to effectively use these technologies. Understanding the factors contributing to these disparities is essential for designing interventions that promote equitable access to digital health tools.

Age is a crucial factor in health care app usage. Younger individuals, with their digital proficiency, are more likely to adopt health care apps compared to older adults [5]. However, older adults, who could significantly benefit from these apps due to higher health care needs, often encounter barriers such as lack of digital literacy and skepticism toward technology [6]. This underscores the need for tailored strategies to bridge this digital divide and encourage app usage among older populations—a key disparity in health care app usage.

Gender differences also play a role in health care app adoption and usage. Studies have shown that men and women exhibit different health-seeking behaviors and preferences for digital health tools [7]. Women are more engaged in web-based health information searching, driven by social motives and enjoyment, while men are more open to virtual patient-physician relationships. Understanding these gender-based preferences can help in developing more gender-sensitive digital health solutions.

Education and income levels are significant predictors of health care app usage. Higher education levels are associated with better health literacy and greater comfort with digital technologies, leading to higher adoption rates of health care apps [8]. Similarly, individuals with higher income levels are more likely to own smartphones and have access to reliable internet, facilitating the use of health care apps [9]. Addressing these socioeconomic barriers is crucial for ensuring digital health tools reach and benefit all population segments.

Although prior studies [10-12] have investigated individual factors associated with health care app usage, such as age-related technology adoption or socioeconomic influences on digital access, existing research remains fragmented. Many studies [10,11] are context-specific, target narrow population groups, or fail to systematically compare findings across demographic and socioeconomic dimensions. Moreover, several studies [11,12] do not provide integrated frameworks that can inform policy or app design across diverse populations.

This systematic scoping review addresses these gaps by synthesizing a wide body of empirical literature to map out how demographic (age and gender) and socioeconomic (education and income) factors contribute to disparities in mobile health app usage. Unlike prior reviews [13,14] that focus on usability or technical features of apps, this review is centered on equity and access, offering practical insights for developers, health care providers, and policy makers aiming to promote inclusive digital health adoption.

The potential of health care apps to enhance health outcomes and reduce health care costs underscores the importance of addressing disparities in their usage. By identifying and understanding the factors that influence app adoption and usage, health care providers and policy makers can develop targeted interventions to promote equitable access to digital health tools. This systematic review aims to synthesize existing research on health care app usage disparities, providing a comprehensive understanding of the demographic and socioeconomic factors at play, and thereby, contributing to the development of more effective and equitable health care policies and practices.

### Objectives

This protocol, adhering to PRISMA-P (Preferred Reporting Items for Systematic Review and Meta-Analysis Protocols) guidelines [15] (Multimedia Appendix 1), underpins a systematic review aiming to identify and synthesize empirical studies on health care app usage disparities. The review seeks to inform stakeholders about the demographic and socioeconomic factors influencing app usage, providing insights to improve app accessibility and effectiveness. This review addresses the following research questions (RQs).

RQ1: What are the key demographic and socioeconomic factors associated with health care app usage disparities?

RQ2: How do these factors influence the adoption and utilization of health care apps?

RQ3: What are the barriers to and facilitators of effective use of health care apps?

## Methods

### Eligibility Criteria and Study Characteristics

Studies will be included in this review based on the following inclusion criteria.

1. Study type: peer-reviewed empirical journal papers (quantitative, qualitative, or mixed methods).
2. Language: published in English.
3. Time frame: published between January 1, 2014, and June 17, 2024.
4. Population: studies involving members of the general public as users of health care apps, with data disaggregated or analyzed by demographic (age and gender) or socioeconomic (education and income) variables.
5. Intervention: studies must examine health care app usage and report outcomes or observations stratified by focus on mobile health care apps used for tasks such as appointment scheduling, remote consultations, health monitoring, medication reminders, or self-management.
6. Outcomes: studies that examine usage patterns, barriers, facilitators, disparities, or adoption metrics stratified by demographic or socioeconomic factors.

### Exclusion Criteria

The exclusion criteria were as follows.

1. Review papers, editorials, opinion pieces, conference abstracts, or non–peer-reviewed literature.
2. Studies that focus on clinical efficacy or technical design of apps without reporting usage by demographic or socioeconomic groups.
3. Studies that examine digital health technologies not focused on mobile apps (eg, web portals, SMS text messaging systems).
4. Studies that do not disaggregate findings by demographic or socioeconomic variables.
5. Studies that focus exclusively on provider use of apps (rather than patient or general population use).

### Definition of Key Constructs

Demographic factors refer to the statistical characteristics of human populations, typically used to identify and describe population segments. In the context of this study, demographic factors include age and gender. These factors help describe who is using the health care apps and how usage varies across population groups.

1. Age refers to the chronological number of years a person has lived, often grouped into categories (eg, children, adolescents, adults, older adults) to assess patterns across the lifespan. In health app research, age is a critical factor influencing digital literacy, health needs, and technology adoption behaviors.
2. Gender refers to the socially constructed roles, behaviors, and identities that societies attribute to individuals, typically categorized as male and female. Gender can influence health-seeking behavior, comfort with technology, and preferences in health care app features and communication styles.

Socioeconomic factors relate to an individual’s or group’s social and economic position in the society, often influencing access to resources, services, and technology. For this review, socioeconomic factors include education level and income level. These factors affect both the ability and willingness to adopt health care apps and are central to understanding digital health disparities.

1. Education level refers to the highest degree or level of formal schooling an individual has completed. Common categories include no formal education, primary education, secondary education, and higher education. Education level influences health literacy, digital competence, and engagement with health technologies.
2. Income level refers to the total monetary earnings an individual or household receives within a specific period (typically monthly or annually). It is often classified into low, middle, and high-income brackets based on the national standards. Income level directly affects access to smartphones, data plans, internet connectivity, and affordability of health-related apps or services.

### Information Sources and Search Strategy

Eight databases were searched for this systematic review. These databases are ACM Digital Library, CINAHL, IEEE Xplore, ProQuest Nursing and Allied Health Journals, PubMed/MEDLINE, ScienceDirect, Scopus, and Web of Science. These databases were selected due to their strengths as discipline-specific databases or citation indexes. All authors have institutional access to these databases. Databases were searched on June 17, 2024. See Table 1 for the search queries and the number of retrieved results. Results were limited to include journal papers written in English and published between January 1, 2014, through June 17, 2024. Keywords were searched in Medical Subject Headings (MeSH) fields in health-focused databases, CINAHL, ProQuest Nursing and Allied Health Journals, and PubMed/MEDLINE. In the other databases, keywords were searched in other metadata fields such as title, abstract, and/or keywords. As of June 2025, screening for duplicates and eligibility criteria has not yet occurred.

**Table 1:** Databases, search queries, and number of studies retrieved.

Database name	Search query	Results (N=1100), n
ACM Digital Library	[[Abstract: “mobile health applications”] OR [Abstract: “digital health”] OR [Abstract: eHealth] OR [Abstract: mHealth] OR [Abstract: telemedicine]] AND [Abstract: “technology acceptance”]	2
CINAHL	MH^a^ (“access to primary care” OR “health services accessibility”) AND MH (“mobile applications”) AND (“digital health” OR telemedicine)	72
IEEE Xplore	(“All Metadata”: mobile health applications” OR “All Metadata”: digital health OR “All Metadata”: eHealth OR “All Metadata”: mHealth OR “All Metadata”: telemedicine) AND (“All Metadata”: technology acceptance)	20
ProQuest Nursing and Allied Health Journals	mainsubject.Exact (“access to primary care” OR “health services accessibility”) AND mainsubject.Exact (“mobile applications”) AND (“digital health” OR telemedicine))	1
PubMed/MEDLINE	(((“access to primary care” OR “health services accessibility”[MeSH^b^ Terms]) AND (“mobile applications”[MeSH Terms])) AND (“digital health” OR “telemedicine”[MeSH Terms]))	6
ScienceDirect	(“mobile health applications” OR “digital health” OR eHealth OR mHealth OR telemedicine) AND (“technology acceptance”)	39
Scopus	Article title, abstract, keywords: (“mobile health applications” OR “digital health” OR eHealth OR mHealth OR telemedicine) AND (“technology acceptance”)	472
Web of Science	Topic (“mobile health applications” OR “digital health” OR eHealth OR mHealth OR telemedicine) AND Topic (“technology acceptance”)	488

^a^MH: CINAHL Exact Subject Headings.

^b^MeSH: Medical Subject Headings.

### Study Records

#### Data Management

All 1100 studies retrieved were imported into the Zotero reference management system. After merging the studies, they will be exported to a Microsoft Excel spreadsheet to remove duplicates, screen unique papers, and select eligible papers. Relevant data characteristics (eg, author names, year of publication, type of study) will be extracted by 2 reviewers and stored in a shared OneDrive folder for systematic analysis and synthesis.

#### Selection Process

Two authors will independently screen all the retrieved records at both the titles/abstracts and full-text stages to ensure methodological rigor and minimize selection bias. Each study will be assessed against the predefined eligibility criteria. The studies that are not excluded will be saved to a separate folder. Subsequently, any discrepancy between the 2 reviewers will be resolved through a tie-breaker process, where a third reviewer is consulted to make the final decision. This dual independent screening process aligns with standard practices in systematic reviews to ensure objectivity and reduce the risk of bias in the study selection process. The final list of the included studies will undergo a collective review to confirm their inclusion in the systematic analysis before proceeding to data extraction and synthesis.

#### Data Collection Process

Data will be extracted using standardized forms by 2 independent reviewers. Key information will be recorded, including study characteristics, population demographics, socioeconomic factors, and app usage metrics. When writing the systematic review, it will be essential to ensure that the data extracted by the independent reviewers are meticulously cross-checked for accuracy and consistency. Any discrepancies between the reviewers will be resolved before moving on. This rigorous approach ensures the reliability of the data, which forms the foundation of the systematic review’s conclusions. Furthermore, the analysis will include a detailed synthesis of the findings, highlighting patterns, correlations, and potential causative factors observed in the data.

#### Quality Appraisal and Risk of Bias Assessment

To ensure the reliability of our systematic review, we will conduct a quality appraisal and risk of bias assessment by using the Mixed Methods Appraisal Tool (MMAT) for evaluating the methodological quality and risk of bias of all the included studies. MMAT is designed to evaluate a broad range of empirical studies, including qualitative, quantitative, and mixed methods designs [16]. Reviews that incorporate diverse study types are increasingly used to examine complex health interventions and outcomes. However, assessing methodological quality across such heterogeneous designs presents a challenge. MMAT was specifically developed to address this issue and has been positively evaluated by researchers for its adaptability and practical usefulness [17].

Two reviewers will independently assess each study to maintain objectivity. Any disagreements will be resolved through discussion or consulting a third reviewer if necessary. We will evaluate the studies based on several key factors: the appropriateness of the research design, the rigor of the data collection and analysis, and the clarity of the results. We will also consider potential biases such as selection bias, performance bias, detection bias, and attrition bias. The results of these assessments will be summarized in a table, highlighting the strengths and weaknesses of each study.

#### Data Analysis and Synthesis

We will use descriptive statistics to summarize the key characteristics of the included studies. This includes study design, population demographics, socioeconomic factors, and health care app usage metrics. They will provide an overview of the scope and variation across the included studies. We will apply standardized summary techniques to ensure consistency in data reporting. First, we will use frequency tables for categorical variables such as gender and app type. Second, we will use means or medians with ranges for continuous variables such as age. Third, we will categorize income and education levels into comparable brackets across studies.

Subgroup patterns will be explored descriptively across predefined categories to identify meaningful variations in usage trends, including (1) app type (telemedicine, medication reminders, fitness, chronic disease management), (2) age group (18-34, 35-49, 50-64, 65+ years old), (3) gender (male, female, other where reported), (4) education level (less than high school, high school graduate, college degree, postgraduate), and (5) income level (<US $25,000, between US $25,000 and US $74,999, >US $75,000).

Due to the diversity in the study characteristics, a structured narrative synthesis will be employed to integrate findings and highlight key disparities. Variations across studies will be organized thematically by subgroup characteristics and usage metrics.

## Results

**Figure 1:**
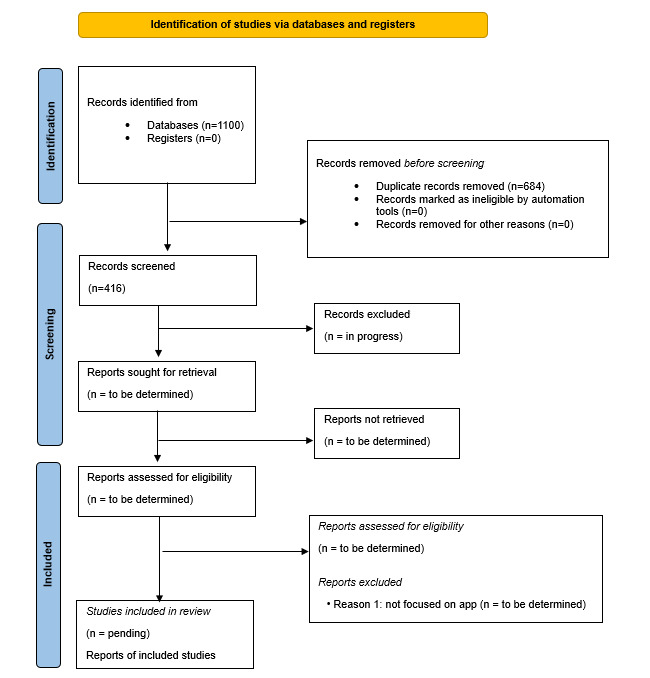
Preliminary PRISMA (Preferred Reporting Items for Systematic Review and Meta-Analysis) flow diagram.

This study was initiated in May 2024, with preliminary data collection starting in June 2024. We anticipate completing data analysis by August 2025. The final results are expected to be published by late 2025. As of May 2025, study deduplication has been completed. From an initial pool of 1100 studies retrieved from 8 databases, 684 duplicates were removed, resulting in 416 unique papers. Title and abstract screenings are currently underway. Full-text screening and eligibility assessment are expected to be completed by November 2025. Figure 1 shows the preliminary PRISMA 2020 flow diagram [18] summarizing the study selection process to date.

## Discussion

### Overview

Recent studies [19-21] have reinforced the urgency of addressing digital health disparities—particularly those amplified during the COVID-19 pandemic. Socioeconomic status continues to be a major determinant of digital health adoption, with lower-income groups and rural populations experiencing persistent barriers to access [22]. Although the pandemic accelerated overall adoption, vulnerable groups, especially those with limited digital literacy or financial resources, remain underserved [22,23]. Age and usability also play a critical role. As highlighted by Gomez-Hernandez et al [24], older adults face challenges in navigating mobile health apps due to visual, cognitive, and motor limitations. Their systematic review provides design guidelines such as simplified interfaces and training support that could significantly improve adoption rates among aging populations.

Gender emerged as a contextual but inconsistently significant factor in digital health adoption. Some studies found that women were more likely to engage with health information online and use digital tools both before and during the COVID-19 pandemic [22], while others reported no significant gender-based differences [25]. A third review acknowledged gender-related disparities in some cases but did not treat gender as a core analytic variable [24]. These mixed findings suggest that gender may interact with other social determinants and deserves further investigation in future research. Tuitert et al [22] demonstrated through a longitudinal study that pandemic-related adaptations helped reduce usage gaps among certain groups, including less-educated individuals. However, inequities based on income and digital access persisted, highlighting the need for ongoing interventions.

Finally, the broader systemic challenges in digital health equity must be acknowledged. As Yao et al [23] emphasize, barriers related to digital literacy, technological infrastructure, and socioeconomic status must be systematically addressed through inclusive design and targeted policies to prevent further marginalization. Together, these studies underscore the need for a comprehensive synthesis of empirical evidence. By identifying and analyzing demographic and socioeconomic drivers of health care app usage disparities, this review aims to support the development of inclusive technologies and equitable digital health strategies that can be adopted across diverse populations.

The findings of this systematic review are expected to provide significant insights into the disparities in health care app usage, influenced by demographic and socioeconomic factors. As health care increasingly shifts toward digital platforms, understanding these disparities is crucial for ensuring equitable access and utilization of health care services. The results will inform stakeholders, including health care providers, policy makers, and app developers, on how to address these disparities and enhance the accessibility and effectiveness of health care apps.

### Implications for Practice

Identifying the specific demographic and socioeconomic factors influencing health care app usage can help design targeted interventions. For instance, if younger individuals are more likely to use these apps, educational campaigns can be tailored for older adults to increase their engagement. Similarly, understanding the impact of education and income levels can guide the development of more user-friendly and affordable app features, ensuring that these digital health tools are accessible to a broader population.

### Policy Recommendations

This review’s findings will be critical for policy makers aiming to reduce health disparities. Policies that promote digital literacy and provide subsidies or financial incentives for using health care apps could be implemented. Furthermore, regulations ensuring the privacy and security of health data are essential to building trust and encouraging app adoption among various demographic groups.

### Technological Considerations

App developers can use the insights from this review to improve the design and functionality of health care apps. Features that cater to the specific needs of different demographic groups, such as language options, simplified user interfaces, and culturally relevant content, can enhance user experience and adoption rates. Additionally, incorporating feedback mechanisms within apps can help developers continuously improve their products based on user experiences and preferences.

### Limitations

This systematic review may face several limitations. First, the restriction to studies published in English and within the past decade might exclude relevant studies published in other languages or before 2014. Second, the reliance on self-reported data in many studies could introduce bias. Third, although the search strategy covered a broad range of terms related to digital health, access, and technology acceptance, it may have benefited from the inclusion of additional terms aligned with the study’s focus on disparities. Keywords such as technology adoption, equity, inequity, and usage could enhance the comprehensiveness of future searches, helping to capture studies that address health disparities and user behavior. Future research should consider these limitations to provide a comprehensive understanding of health care app usage disparities, thereby enhancing the scope of future reviews.

### Conclusion

In conclusion, this systematic review aims to illuminate the disparities in health care app usage, offering practical insights for improving digital health equity. By addressing the demographic and socioeconomic factors that influence app usage, stakeholders can develop more inclusive and effective digital health strategies. The anticipated results will contribute to the growing body of knowledge in digital health and support the development of targeted interventions to bridge the digital divide in health care access.

## Appendix

Multimedia Appendix 1 PRISMA-P 2015 checklist.

## Abbreviations

MeSH: Medical Subject Headings
MMAT: Mixed Methods Appraisal Tool
PRISMA-P: Preferred Reporting Items for Systematic Review and Meta-Analysis Protocols
RQ: research question

## Data Availability

As this is a protocol, no data have been generated yet. Upon completion of the review, the extracted dataset will be made available upon reasonable request from the corresponding author or deposited in an open-access repository where appropriate.
